# Accumulation of interspersed and sex-specific repeats in the non-recombining region of papaya sex chromosomes

**DOI:** 10.1186/1471-2164-15-335

**Published:** 2014-05-04

**Authors:** Jong-Kuk Na, Jianping Wang, Ray Ming

**Affiliations:** Department of Plant Biology, University of Illinois at Urbana-Champaign, Urbana, IL 61801 USA; Molecular Breeding Division, National Academy of Agricultural Science, RDA, Suwon, 441-701 Republic of Korea; Department of Agronomy, Genetics Institute, Plant Molecular and Cellular Biology program, University of Florida, Gainesville, FL 32610 USA; FAFU and UIUC-SIB Joint Center for Genomics and Biotechnology, Fujian Agriculture and Forestry University, Fuzhou, Fujian 350002 China

**Keywords:** Bacterial artificial chromosome (BAC), *Carica papaya*, Hermaphrodite-specific region of the Y chromosome (HSY), Recombination suppression, Repetitive sequence, Sex-specific repeat

## Abstract

**Background:**

The papaya Y chromosome has undergone a degenerative expansion from its ancestral autosome, as a consequence of recombination suppression in the sex determining region of the sex chromosomes. The non-recombining feature led to the accumulation of repetitive sequences in the male- or hermaphrodite-specific regions of the Y or the Y^h^ chromosome (MSY or HSY). Therefore, repeat composition and distribution in the sex determining region of papaya sex chromosomes would be informative to understand how these repetitive sequences might be involved in the early stages of sex chromosome evolution.

**Results:**

Detailed composition of interspersed, sex-specific, and tandem repeats was analyzed from 8.1 megabases (Mb) HSY and 5.3 Mb corresponding X chromosomal regions. Approximately 77% of the HSY and 64% of the corresponding X region were occupied by repetitive sequences. *Ty3-gypsy* retrotransposons were the most abundant interspersed repeats in both regions. Comparative analysis of repetitive sequences between the sex determining region of papaya X chromosome and orthologous autosomal sequences of *Vasconcellea monoica*, a close relative of papaya lacking sex chromosomes, revealed distinctive differences in the accumulation of *Ty3-Gypsy*, suggesting that the evolution of the papaya sex determining region may accompany *Ty3-Gypsy* element accumulation. In total, 21 sex-specific repeats were identified from the sex determining region; 20 from the HSY and one from the X. Interestingly, most HSY-specific repeats were detected in two regions where the HSY expansion occurred, suggesting that the HSY expansion may result in the accumulation of sex-specific repeats or that HSY-specific repeats might play an important role in the HSY expansion. The analysis of simple sequence repeats (SSRs) revealed that longer SSRs were less abundant in the papaya sex determining region than the other chromosomal regions.

**Conclusion:**

Major repetitive elements were *Ty3-gypsy* retrotransposons in both the HSY and the corresponding X. Accumulation of *Ty3-Gypsy* retrotransposons in the sex determining region of papaya X chromosome was significantly higher than that in the corresponding region of *V. monoica*, suggesting that *Ty3-Gypsy* could be crucial for the expansion and evolution of the sex determining region in papaya. Most sex-specific repeats were located in the two HSY expansion regions.

**Electronic supplementary material:**

The online version of this article (doi:10.1186/1471-2164-15-335) contains supplementary material, which is available to authorized users.

## Background

Papaya (*Carica papaya* L.) is a major tropical fruit crop, and the only species in the genus *Carica*. Papaya shared a common ancestor with *Arabidopsis* approximately 72 million years ago. Its short juvenile phase of 3 to 4 months, continuous flowering, short generation time of 9 months, and small genome size of 372 Mb [1] make papaya a promising model for tropical fruit tree genomics [2]. Though the papaya genome size is three times that of *Arabidopsis*, the annotation of papaya’s whole genome sequence revealed that it contains fewer genes than *Arabidopsis*[2], suggesting that the papaya genome might contain significantly more repetitive sequences than the *Arabidopsis* genome.

The *Caricaceae* family consists of 35 species; one monoecious, 32 dioecious, and two trioecious species, providing an invaluable system for studying plant sex determination. *Vasconcellea Monoica* is a monoecious species with no sex chromosomes, whereas all dioecious and trioecious species are likely to have sex chromosomes. Papaya is a trioecious species with three sex phenotypes; female, male, and hermaphrodite. The sex determination of papaya is controlled by a pair of primitive sex chromosomes. Female papaya has homogametic XX chromosomes, whereas male and hermaphrodite plants have heterogametic XY chromosomes. The male and the hermaphrodite have slightly different Y chromosomes, Y for males and Y^h^ for hermaphrodites [3, 4].

The papaya hermaphrodite-specific Y^h^ chromosome (HSY) region occupies approximately 13% of the Y^h^ chromosome [5], and the chromosomal genetic recombination around this region is suppressed [6, 7], a typical feature of sex chromosomes [4]. The suppression of recombination creates conditions that are favorable for the accumulation of deleterious mutations in the non-recombining region of Y^h^ chromosome, and consequently the HSY has evolved in both physical size and gene content to differentiate from the corresponding X [8]. The highly diverged human X and Y chromosomes only share about a dozen pairs of genes in the male specific region of the Y chromosome (MSY). The human Y chromosome is occupied by nearly 95% MSY, and only 5% terminal area, called pseudoautosomal regions, accounting for crossing over with the X chromosome [9]. The human Y chromosome contains a high percentage of repetitive elements and duplicated regions but low gene content [9, 10]. Compared to the human MSY, the papaya HSY is at the early stage of its evolution and occupies only 13% of the Y^h^ chromosome [5], but analysis of HSY bacterial artificial chromosomes (BACs) revealed that the papaya HSY contained significantly higher repeat content [3, 11]. In addition, the sequence analysis of these BACs exhibited a higher content of *Ty3-gypsy* and some *Ty1-copia* retroelements, which are normally abundant near the centromeric region.

Although it is well known that the recombination suppression of homologous sex chromosomes causes the accumulation of repetitive sequences, little is known about the feature of sex-specific repeats in plants. Sex-specific markers are important for determining the presence of sex chromosomes [12]. In date palm (*Phoenix dactylifera*), the presence of sex chromosomes was verified by the identification of sex-specific DNA markers [13]. In hop (*Humulus lupulus* L.), inter simple sequence repeat (ISSR) markers were identified as sex-specific markers [14]. To date, dozens of sex-specific markers have been identified in various plant species and they are mostly used to support the presence of sex chromosomes [15]. If the Y chromosome is degenerated progressively, then sex-specific repeats could be a very useful marker to examine the lineage of Y chromosomes among plant species and perhaps they are useful to understand duplication events occurred in a given Y chromosome. Recently, four Y-specific satellite DNA families, RAYSI, RAE180, RAYSI-S, and RAYSI-J, were identified from *Rumex acetosa* and used successfully as the references to examine the degeneration of the Y chromosome among the genus *Rumex*[16, 17]. Therefore, identification of sex-specific repeats and analysis of their sequence features in papaya can provide valuable genomic resources for unraveling genetic lineages of sex chromosomes among dioecious and trioecious species in the *Caricaceae* family and for revealing the roles that sex-specific repeats play in the sex chromosome evolution. As for agricultural aspects of papaya or other fruit crops with different sex types, sex-specific repeats can be used to develop molecular markers that distinguish plant sex types at the seedling stage.

The insertions of transposable elements are believed to be one of the earliest triggers that cause the suppression of recombination [18]. Since papaya sex chromosomes are believed to be at an early stage of evolution, the information from papaya repetitive sequence analysis could be used to test whether such insertions of transposable elements are indeed a cause for the recombination suppression by out-crossing with monoecious *V. monoica*. Here, we report not only the detailed repetitive sequence features of the newly sequenced papaya HSY and the corresponding X, but also the comparison of repetitive sequence features between the papaya sex determining region and the orthologous autosomal region in *V. monoica*, which has no sex chromosomes [19], to provide insights into papaya sex chromosome evolution and their sequence features. As expected, the HSY is highly abundant with interspersed repeats compared to the corresponding X chromosome and the papaya genome. A new search of interspersed repeats in the given sequences enabled the identification of 36 new repeats with 21 of them being sex-specific repeats, which probably could be used as a reference for analysis of Y chromosomes among the other species in the *Caricaceae* family.

## Results

### Composition of interspersed repeats in the sex determining region of papaya

To examine repetitive sequences in both the HSY and the corresponding X, the sequences were masked by RepeatMasker using a customized repeat database as a library consisting of Repbase, TIGR repeat data, and papaya repeats [20]. Results showed that the interspersed repeats occupied approximately 77% of the HSY (6,226,262 bp), 64% of the corresponding X (3,379,825 bp), and only 20.9% of *V. monoica* (Table 1). Among all interspersed repeats identified, the retroelements were the most abundant repeats, 64%, 54%, and 16% in the HSY, the corresponding X, and *V. monoica*, respectively. These retroelements accounted for the vast majority of all identifiable interspersed repeats and only a small fraction (< 1%) of the interspersed repeats were DNA transposons in the HSY, the corresponding X, and *V. monoica* (Table 1). Therefore, it is likely that the majority of unclassified interspersed repeats (13.5% in the HSY and 9.6% in the corresponding X) could be classified into retroelements if they could be annotated (Table 1).

**Table 1:** **Interspersed repeats in the sex determining region on papaya sex chromosomes**

Repeat class/family	HSY (8062184 bp)				Corresponding X (5298217 bp)				***Vm*** X (1079651 bp)			
	Known repeats		Known plus papaya repeats		Known repeats		Known plus papaya repeats		Known repeats		Known plus papaya repeats	
	Length occupied (bp)	Percentage of sequence (%)	Length occupied (bp)	Percentage of sequence (%)	Length occupied (bp)	Percentage of sequence (%)	Length occupied (bp)	Percentage of sequence (%)	Length occupied (bp)	Percentage of sequence (%)	Length occupied (bp)	Percentage of sequence (%)
Retroelements	1375153	17.0	5130402	63.6	882569	16.4	2867602	54.1	114116	10.5	178174	16.4
LINEs	560	0.0	50037	0.6	778	0.0	64004	1.2	333	0.0	6351	0.6
LTR elements	1374593	17.0	5080365	63.0	881791	16.4	2803598	52.9	113782	10.5	171823	15.9
Ty1/Copia	180993	2.2	400619	5.0	166009	3.1	332850	6.3	59403	5.5	97554	9.0
Ty3/Gypsy	1065137	13.2	3735520	46.3	616379	11.5	1997877	37.7	32631	3.0	48144	4.4
DNA transposons	1111	0.0	7819	0.1	1256	0.0	4704	0.1	4147	0.4	8863	0.8
En-Spm	81	-	81	0.0	526	0.0	512	0.0	2581	0.2	4513	0.4
MuDR-IS905	-	-	-	-	93	-	93	0.0	402	0.0	901	0.1
Unclassified	193258	2.4	1088041	13.5	57890	1.1	507519	9.6	5876	0.5	39784	3.7
Total interspersed repeats	1569522	19.5	6226262	77.2	941715	17.5	3379825	63.8	124139	11.5	226821	20.9

**Figure 1:**
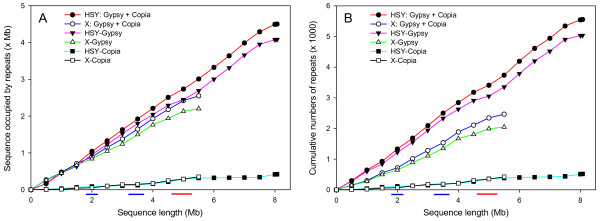
**Cumulative distributions of** ***Ty3-gypsy*** **and** ***Ty1-copia*** **long terminal repeat (LTR) elements in the sex-determining chromosome regions. (A)** The cumulative increase of sequences occupied by *Ty3-gypsy* and *Ty1-copia* LTR elements in hermaphrodite-specific Y (HSY) chromosome region and its corresponding X region. **(B)** The cumulative numbers of *Ty3-gypsy* and *Ty1-copia* LTR elements in the HSY and the corresponding X region. The distance between two dots represents 250 kb. Colored bars at an X-axis denote regions with significantly low repeat contents in the HSY (red) or in the corresponding X region (blue).

Long terminal repeats (LTRs) accounted for more than 97% of all identifiable retroelements in all three sources of sequences and the *Ty3-gypsy* element was the most abundant LTR in the HSY and the corresponding X, whereas *Ty1-copia* element was more abundant in *V. monoica* (Table 1). The number and sequences of *Ty3-gypsy* elements increased notably along the increase of sequence length in the HSY and the corresponding X (Figure 1A and B). To examine the portion of papaya-specific repeats accounting for interspersed repeats, the HSY and the corresponding X sequences were also masked by only known repeats, consisting of Repbase and TIGR repeats excluding papaya repeats. The known repeat content was approximately 19.5% in the HSY; 2% higher than 17.5% in the corresponding X (Table 1). As a result, papaya-specific repeats were at least 57.8% and 46.3% in the HSY and in the corresponding X, respectively.

Although the HSY and the corresponding X were highly occupied by interspersed repeats, there were potential gene rich regions with significantly low repeat accumulation. Two large sequence blocks with scarce or no repeats were detected from 1.8 to 2.2 Mb and from 3.2 to 3.7 Mb in the corresponding X [21], whereas only one large block with low repeat content was found from 4.6 to 5.3 Mb region in the HSY [21].

### Identification of sex-specific repeats in the papaya sex determining region

From the extensive search for sex-specific repeats in the sex determining region, 36 putative sex-specific repeats were identified, 33 from the HSY and three from the corresponding X (Additional file 1: Note 1). In order to determine sex-specific repeats among the 36 newly identified repeats, all repeats were aligned against papaya genome sequences. Among them, 21 repeats were selected as potential sex-specific repeats because they had no match or very low occurrence in the papaya genome (< 10 times; Additional file 2: Table S1). Although the rest of the repeats were present in both the sex determining region and the papaya genome, they were more frequent in the sex determining region (Additional file 2: Table S1). The 36 new repeats occupied approximately 19.9% of the HSY, 12.9% of the corresponding X, and 5.7% of the papaya genome (Table 2). By contrast, the 21 sex-specific repeats accounted for 10.7% of the HSY sequences, 3.5% of the corresponding X, and 0.9% of the papaya genome (Table 2). To test whether papaya and *V. monoica* share any common repeat sequences, we analyzed the accumulation of the sex-specific repeats in *V. monoica* shotgun sequences and the 11 *V. monoica* BAC sequences corresponding to the sex determining region of the X chromosome. Both *V. monoica* genome and the BAC sequence showed much less sex-specific repeat accumulation (Table 2).

**Table 2:** **Accumulation of newly identified repeats from the sex determining region**

Sequence source	All new repeats			Sex specific repeats			Sequence length (bp)
	# of elements	Length occupied (bp)	% of sequence	# of elements	Length occupied (bp)	% of sequence	
HSY	3762	1609173	19.9	1944	866694	10.7	8062184
Corresponding X	1516	692894	12.9	483	188943	3.51	5298217
*Vm* X	12	4779	0.4	7	3783	0.4	1079651
*Cp* genome	47698	21203993	5.7	9245	3436469	0.9	271742010
*Vm* genome	6052	723335	0.3	3469	475808	0.2	245072629

**Figure 2:**
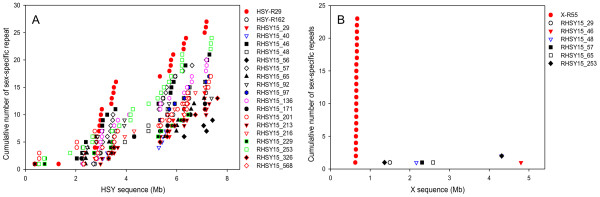
**Cumulative distributions of the sex-specific repeats identified from the sex-determining chromosome regions. (A)** The accumulative number of each sex-specific repeat in the hermaphrodite-specific Y (HSY) chromosome region. **(B)** The accumulative number of each sex-specific repeat in the HSY-corresponding X region. In total, 21 sex-specific repeats were identified from the sex determining region; 20 from the HSY and one from the corresponding X. Most of HSY-specific repeats were located in two regions where the HSY expansion occurred.

To examine the localization of the sex-specific repeats in the sex determining region, all positions aligned with the sex-specific repeats were plotted to their corresponding locations in the HSY (Figure 2A) or in the corresponding X (Figure 2B). Most HSY-specific repeats were located in two regions in the HSY, from 2.0 to 4.0 and 5.0 to 7.5 Mb (Figure 2A), but rarely found in the corresponding X except for X-R55 (Figure 2B), an X-specific repeat. Remarkably, the two regions in the HSY with high HSY-specific repeats were matched to two HSY expansion regions very well [21].

**Figure 3:**
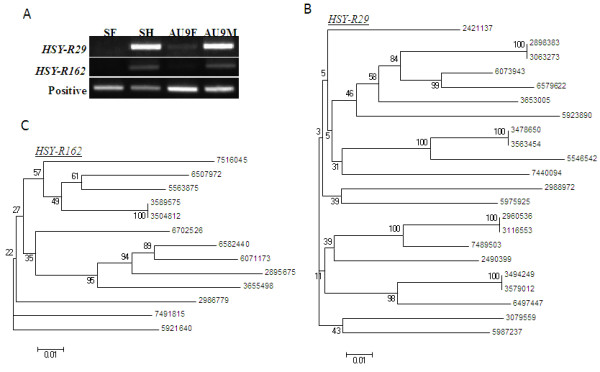
**Identification, validation, and phylogenetic analyses of sex-specific repeats in the sex determining chromosome regions. (A)** Gel image of genomic PCR result from male-specificity test of HSY-R29 and HSY-R162 (SF: SunUp female, SH: SunUp hermaphrodite, AU9F: AU9 Female, AU9M: AU9 male). Phylogenetic analyses of papaya HSY-specific repeats, HSY-R162 **(B)** and HSY-R29 **(C)**. Individual repeat ID represents its proximal location (bp) in the HSY.

Among the 21 potential sex-specific repeats, two HSY-specific repeats, HSY-R29 and HSY-R162, and one X-specific repeat, X-R55, were selected for further analyses. Hermaphrodite specificity of both HSY-specific repeats was confirmed by PCR using genomic DNA samples as templates. Both repeats were confirmed to be present only in SunUp hermaphrodite plants having both the hermaphrodite Y^h^ and X chromosomes, but not in SunUp females having two X chromosomes (Figure 3A). Since all HSY-specific repeats were present in more than 10 copies (Additional file 3: Table S2), it was of interest to examine the phylogenetic relationship among copies of each HSY repeat. Twenty-two aligned sequences to HSY-R29 and thirteen aligned sequences to HSY-R162 were retrieved from the HSY sequences for phylogenetic analysis. Phylogenetic analysis revealed that there was no correlation between distance and sequence identity among copies (Figure 3B and C), which was further confirmed by the Mantel test (Additional file 4: Figure S1). The correlation coefficient of the Mantel test (Rxy) and the one-tailed p-value (rxy-rand ≥ rxy-data) were 0.014 and 0.511 for the HSY-R29 and -0.033 and 0.378 for the HSY-R162, respectively.

**Figure 4:**
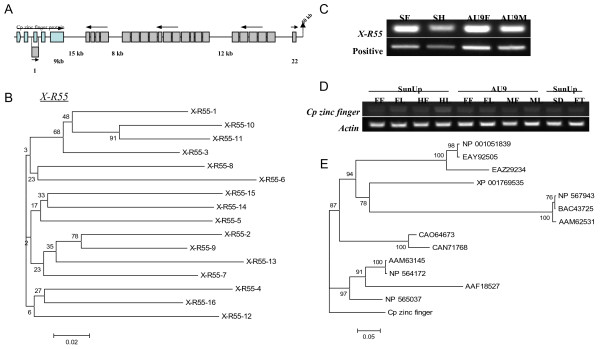
**Association of potential X-specific repeat in the exon duplication of papaya zinc finger protein. (A)** Schematic demonstration of the duplication of X-specific repeat, X-R55, containing the 3rd exon of papaya zinc finger protein (accession ID: EX272522.1). **(B)** Phylogenetic tree of multiple X-R55 copies. The numbers at the root of each branch joining point are boot strap values. **(C)** Gel image of genomic PCR result for testing presence of the X-R55 (SF: SunUp female, SH: SunUp hermaphrodite, AU9F: AU9 Female, AU9M: AU9 male). **(D)** Gel image of RT-PCR result for testing the expression of papaya zinc finger protein in various tissues from SunUp and AU9 papaya (FF: female flower, FL: female leaf, HF: hermaphrodite flower, HL: hermaphrodite leaf, MF: male flower, ML: male leaf, SD: seeds, FT: 50% mature fruit). **(E)**. Phylogenetic tree of papaya zinc finger protein (*Cp* zinc finger) with homologous proteins from other plant species with accession ID in NCBI.

The potential X-specific repeat, X-R55, appeared 22 times (> 100 bp) only in the corresponding X, but not in the HSY and papaya genome sequences (excluding X chromosome). The copies of the X-R55 repeat were present within a small range with ~50 kb (Figure 4A) in the corresponding X, and the repeat size ranged from 109 to 306 bp. Except for the two copies at both ends, the rest of the 20 copies were oriented in the same direction and tandemly repeated in three separate repeat blocks, apart from each other by about 8 to 15 kb (Figure 4A). The first repeat block near a zinc finger protein consisted of 4 tandem repeats, the second block of 11, and the third block of 5 (Figure 4A). Among the 22 copies of X-R55 repeat, the 17 copies longer than 200 bp were used for phylogenetic analysis. Similar to the result of phylogenetic analysis of two HSY-specific repeats, the distance among individual copies did not show correlation to the sequence similarity (Figure 4B, Additional file 4: Figure S1). An interesting feature of the X-R55 repeat was that it showed very high sequence identity (91%) with the third exon of a potential *Carica papaya* (*Cp*) zinc finger protein nearby (Figure 4A), whose expression was confirmed by an expressed sequence tag (GB: EX272522.1). If the X-R55 repeats originated from the third exon of the *Cp* zinc finger protein, the second repeat block could be the most recently duplicated, on the basis of phylogenetic analysis (Figure 4B). The presence of the X-R55 repeat was confirmed by PCR (Figure 4C). The expression of the *Cp* zinc finger protein was examined by RT-PCR and detected in all sex types of flowers and leaf tissues from ‘SunUp’ and ‘AU9’ papayas, and also in seed and half ripened fruit of ‘SunUp’ (Figure 4D). Phylogenetic analysis revealed that the *Cp* zinc finger protein was closely related to *Arabidopsis* zinc finger gene (NP_565037) (Figure 4E).

### Accumulation of SSRs in the sex determining region

**Figure 5:**
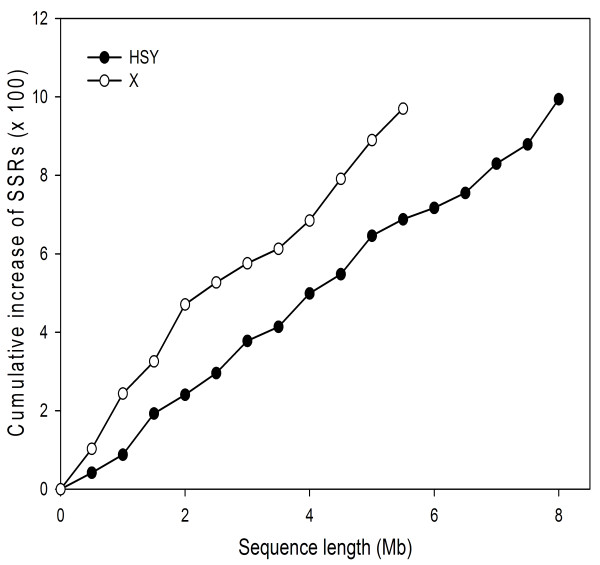
**Cumulative distributions of simple sequences repeats (SSRs) in the sex determining chromosome regions.** SSRs with a length greater than 12 nucleotides, motif lengths of 2 to 6 bp, and a minimum of 5 repeats, were detected from the HSY and the corresponding X sequences. Number of total SSRs identified from each 0.5 Mb was plotted at the corresponding positions on the HSY and the corresponding X.

Accumulation of repetitive sequences is one of the key elements for the degeneration of sex chromosomes. Accordingly, high repetitive sequence accumulation was observed in papaya HSY and in the corresponding X compared to that of papaya genome [2, 21]. However, not only interspersed repeats but also tandem DNA repeats were accumulated in sex chromosomes [22]. Simple sequence repeats (SSRs) of the short tandem DNA repeats normally originate from slippage during DNA replication. Therefore, SSRs were examined in the HSY and the corresponding X (Figure 5 and Table 3). The SSR densities were much lower in the HSY (one per 8.1 kb) than in the corresponding X (one per 5.4 kb) and the papaya genome (one per 3.2 kb). On the other hand, SSR densities of *V. monoica* BAC sequences were even higher (one per 2.3 kb) than papaya genome (Table 3). SSRs have been categorized into two classes, class I and class II. Class I includes hypervariable SSRs ≥ 20 bp, whereas class II consists of less variable SSRs ≥ 12 bp and < 20 bp [23]. Class I and class II SSR densities were lower either in the HSY or in the corresponding X compared to those in the papaya genome (Table 3). Class I and Class II SSR densities in *V. monoica* BAC sequence were comparable to those in *V. monoica* shotgun genome (Table 3). Class I SSR densities in the corresponding X, *V. monoica* BACs and shotgun sequence, and papaya genome were approximately two-fold less than that of class II SSR, but much less in the HSY compared to the rest. SSR density of di-nucleotide SSR units were similar between papaya genome and *V. monoica* shotgun or BAC sequences, but SSR density of tri-nucleotide SSR units in *V. monoica* genome was significantly higher than that of the papaya genome (Table 4). Therefore, it was evident that SSR frequency in the sex determining region was lower than that in papaya genome and *V. monoica* genome.

**Table 3:** **Distribution of SSR classes identified in difference sources of sequences**

Sequence sources	Size (Mb)	Class I SSR		Class II SSR		Total SSR	
		Number	Density (Kb/SSR)	Number	Density (Kb/SSR)	Number	Density (Kb/SSR)
HSY	8.1	248	32.5	748	10.8	996	8.1
Corresponding X	5.3	317	16.7	660	8.0	977	5.4
*Vm* X	1.1	173	6.2	289	3.7	462	2.3
*Cp* genome	271.7	28799	9.4	55162	4.9	83961	3.2
*Vm* genome	245.1	29364	8.3	69346	3.5	98710	2.5

**Table 4:** **SSR distribution by SSR unit size**

Unit size		HSY		Corresponding X		***Vm*** X		***Cp*** genome		***Vm*** genome
	Number	Density (Kb/SSR)	Number	Density (Kb/SSR)	Number	Density (Kb/SSR)	Number	Density (Kb/SSR)	Number	Density (Kb/SSR)
2	742	10.9	769	6.9	331	3.3	66431	4.1	68405	3.6
3	212	38.0	171	31.0	119	9.1	13866	19.6	27189	9.0
4	15	537.5	11	481.7	4	269.9	2248	120.9	2070	118.4
5	24	335.9	19	278.9	4	269.9	1062	255.9	463	529.3
6	3	2687.4	7	756.9	4	269.9	354	767.6	583	420.4

## Discussion

In this study, we characterized the detailed genomic structure of the papaya sex determining region by analyzing the interspersed and short tandem repeat distribution and identifying potential sex-specific repeats. Analysis of sex-specific repeats revealed that the accumulation and distribution of these repeats have a very close relationship with the expansion of the sex determining region, implying that sex-specific repeats may play crucial roles in the differentiation of sex chromosomes. In addition, the corresponding X was compared to orthologous autosomal sequences of *V. monoica*, revealing that the expansion of the papaya sex determining region is associated with increased frequency of *Ty3-gypsy* retroelements.

### Distribution of repetitive sequences

Y chromosomes are featured by degeneration, duplication, and abundance of repetitive sequences due to a non-recombining property. The HSY sequences on the papaya Y^h^ chromosome were occupied by higher repetitive sequences, compared to its X counterpart [2, 21]. The average repeat content of the HSY was approximately 77%, 13% higher than the 64% of the corresponding X (Table 1). These numbers were different from what we reported previously [2, 21], which was caused by the analysis of all 5.3 Mb X sequences including 1.8 Mb Knob 1 sequences that were not included in the previous report. The high accumulation of repetitive DNA sequences was shown in ancient Y chromosomes in human [24] and *Drosophila melanogaster*[25], also in the nascent Y chromosome in *Drosophila Miranda*[26] and *Silene latifolia*[22]. Repeat contents of the HSY and the corresponding X increased dramatically when masked by a repeat library containing both papaya and public repeat sequences, compared to the repeat contents of 19.5% of the HSY and 17.5% of the corresponding X when both sequences were masked by only publicly available known repeat sequences (Table 1), indicating that the majority of repeats accumulated in the sex determining regions are most likely papaya-specific [20]. It is worth noting that the conserved repetitive sequences in the HSY and its X counterpart were more than the genome-wide average of 14% [2] and also higher than the repeat content in *V. monoica*, which has no sex chromosomes, reinforcing the notion that increased repetitive sequences are a feature of the sex determining region.

*Ty3-gypsy* elements were highly accumulated in the sex determining region and accounted for 46.3% of the HSY and 37.7% of the corresponding X (Table 1 and Figure 1). The *Ty3-gypsy* content of the HSY was ~8% lower than that the previous study estimated from sequences of seven HSY BACs where it was 54% [2], which might be due to uneven distribution of *Ty3-gypsy* elements throughout the HSY. On the other hand, the *Ty1-copia* elements were less abundant compared to *Ty3-gypsy* in both the HSY and the corresponding X (Table 1 and Figure 1). *Ty1-copia* content in the corresponding X was 1.3% higher than that in the HSY (Table 1), suggesting that *Ty1-copia* elements were not a major contributor to repeat accumulation in both the HSY and the corresponding X. This result is different from the retroelement accumulation in *S. latifolia* Y chromosome where *Ty1-copia* elements are more abundant than *Ty3-gypsy* elements [27]. It could be due to the incomplete sequences of *S. latifolia* Y chromosome or the feature of the very early evolutionary stage of homomorphic Y^h^ chromosome in *C. papaya* compared to heteromorphic Y chromosome such as in *S. latifolia*, *Rumen acetosella*, and *Marchantia polymorpha*.

### Decreased SSR frequency in the sex determining region

SSR density was significantly lower in the HSY and in the corresponding X compared to that in papaya or *V. monoica* genomes (Table 3) due to the increase of the overall repetitive sequence and the decrease of gene content [21]. These results suggest that the HSY is less vulnerable to mutation caused by replication slippage compared to other chromosome regions to maintain its unique sequence feature. Class I and class II densities in *V. monoica* BAC sequences were comparable to those in *V. monoica* shotgun genome, whereas those densities in the sex determining region were much lower than those in the papaya genome (Table 3), indicating that low SSR density in the HSY and the corresponding X was most likely caused by the process of the evolution of papaya sex chromosomes accompanied by the insertion of repetitive sequences. The density of Class I SSR (longer than class II SSR) in the HSY was lower than the rest, suggesting that the longer SSRs might be more susceptible to degeneration in the HSY.

### Sex-specific repeats

Papaya sex types are determined by a small non-recombining region of recently evolved sex chromosomes [2]. The suppression of recombination in the HSY accompanies the accumulation of repetitive sequences and chromosomal rearrangements. These changes might ultimately result in the evolution of sex-specific repeats and the differentiation of sex chromosomes from their ancestral autosomes. Phylogenetic analysis and Mantel test of three sex-specific repeats, HSY-R29, HSY-R162, and X-R55, revealed that the distance and sequence similarity among copies of each repeat had no correlation (Figure 3B and C, Additional file 4: Figure S1), indicating that the insertion of repeats occurred either in a random manner regardless of the physical distance between original and new target sites or rearrangements occurred after tandem duplications. The HSY-R29 and HSY-R162 did not show any similar sequence match from the NCBI nucleotide database and TIGR plant repeat database (http://plantrepeats.plantbiology.msu.edu/). However, many HSY-R29 flanking sequences (~500 bp) showed similarity to chloroplast DNA of papaya and other plant species (data not shown), suggesting that the possible origin of HSY-R29 might be associated with chloroplast DNA insertions. DNA fragments transferring from organelles are not rare. For example, there is over 100 kb chloroplast DNA in rice chromosome 10 [28]. The papaya genome also contains nearly 1 Mb chloroplast DNA [2]. The papaya HSY accumulated a staggering amount of chloroplast DNA due to its lack of recombination with the corresponding X chromosome. The chloroplast DNA insertion could be another means of sex chromosome evolution.

Several sex-specific repeats were identified in other plant species, such as the RAYSI - III family in the plant *Rumex acetosa*[17, 29], MADC1 in *Cannabis sativa*[30], and the tandem Y-specific DNA repeats in *Marchantia polymorpha*[31]. The RAYSI-III family is satellite DNAs and MADC1 is homologous to LINE-like retrotransposons with a site-specific accumulation of the long arm of the Y chromosome [30]. Like the Y-specific repeats in *M. polymorpha*[31], the HSY-R29 and HSY-R162 were identified as sex-specific repeats and exhibited no similarity to any known repetitive sequences such as retroelements or satellite DNAs, indicating that these repeats are specific to the sex determining region of the papaya genome.

The Y-specific repeats of *M. polymorpha* are not only tandemly duplicated, but also contain male-specific genes [31]. In humans, it was also reported that the active gene could be multiplied as a result of tandem duplications and large sequence inversions, such as the AZFc region of the Y chromosome [32, 33] and the ZNF91 gene family in chromosomes 19 and 7 [34, 35]. In this study, we identified a potential X-specific repeat X-R55, which contained the third exon of a papaya zinc finger protein (Figure 4A). The tandem duplication of X-R55 was quite similar to the ZNF91 subfamily of primate-specific zinc finger genes, consisting of large gene clusters with some dysfunctional copies [34]. Another interesting feature of the ZNF91 gene family was that the large gene clusters are located near the centromere of chromosomes 19 and 7 [34]. In papaya, gene duplication was reported [20], and some of those genes may be clustered as similar to the ZNF 91 gene family. Nevertheless, this finding raises questions about whether the X-R55 repeats are located near the centromere of papaya X chromosome and whether the duplication of the X-R55 passed through a similar process as ZNF91 after duplication, such as loss of function and alternative splicing. These questions remain to be further investigated.

## Conclusions

We analyzed repetitive sequences and sex-specific repeats accumulated in the HSY and its X counterpart of papaya sex chromosomes. The sequences of the HSY and the corresponding X were highly repetitive as 77% of the HSY and 64% of the X counterpart sequences were found to be repetitive, of which the major repeat element was *Ty3-gypsy*. The HSY and its X counterpart contained sex-specific repeats, including 20 HSY-specific repeats and one X-specific repeat. Most HSY-specific repeats exhibited accumulation at specific locations in the HSY, where the HSY expansions took place. The HSY expanded at an accelerated pace compared to its X counterpart and the HSY-specific repeats contributed to its rapid expansion.

## Methods

### DNA sequences

The sequences of 13.4 Mb consisting of 8.1 Mb of HSY and 5.4 Mb of the corresponding X chromosome [21, 36] were used to examine repetitive genomic features and SSR distribution. In addition, a 245 Mb of *V. monoica* genomic shotgun sequences and a 1.1 Mb of 11 *V. monoica* BAC sequences [19] were used to compare accumulation and distribution of repetitive sequences and SSRs with those in the HSY, the corresponding X, and papaya genome.

### Tandem repeats

A perl program, **MI**cro**SA**tellite identification tool (MISA; http://pgrc.ipk-gatersleben.de/misa/download/misa.pl), was used to mine SSRs in the given sequences. SSRs with a length greater than 12 nucleotides, motif lengths of 2 to 6 bp, and a minimum of 5 repeats were detected and analyzed.

### Interspersed repeats analysis against known repeat databases

The repeat library was generated by combining Repbase [37], TIGR plant repeats (ftp://ftp.tigr.org/pub/data/ TIGR_Plant_Repeats), and papaya repeats [20]. For analyzing the repeat composition in the HSY and the corresponding X, RepeatMasker (http://www.repeatmasker.org) was used to analyze the repeat composition in the HSY and the corresponding X using the repeat library with default settings.

### Identification of new repeats in the sex determining region

To identify new repeats, the sequences of the HSY and the corresponding X were first run on RepeatScout [38] to generate putative repeat sequences. Then, the resulting repeats were run on RepeatMasker (http://repeatmasker.org) to mask the HSY or the corresponding X sequences and to screen the repeats with the occurrence of more than 10 times and aligned length longer than 100 bp. Next, the non-redundant repeats passing above criteria were determined as new repeats by comparing them to previously identified papaya repeats from female papaya genome sequence [20] using CD-HIT software [39] with a cutoff of 70% similarity. Finally, the new repeats were blasted against the HSY and the corresponding X sequences using Standalone BLAST software (NCBI) and screened based on the following more stringent criteria: 1) at least a 50% alignment over a consensus sequence, 2) occurrence of at least 10 times, and 3) an aligned region with at least 100 bp and > 75% identity. The repeats that met these criteria were re-screened with property of less than 10 hits in the papaya genome in order to obtain potential sex-specific repeats. Clustalw [40] and MEGA [41] software were used for phylogenetic analysis of the repeats.

### PCR for sex-specific repeats

Samples from SunUp female, SunUp hermaphrodite, AU9 female, and AU9 male were used to isolate genomic DNAs as described by K Edwards, C Johnstone and C Thompson [42] with slight modifications. PCR was carried out with 5 ng of DNA as a template with the following primer sets: HSY-R162 (Forward: 5′-TTTGTTCTCCTCTCAGCTTGC-3′; Reverse: 5′-GCCATACACGTAATGGGAAAA3′), HSY-R29 (Forward: 5′-GAAACCCATGCGAAGGAATA-3′; Reverse: 5′-TGGGATTCTTTTTGGGTCAG), and X-R55 (Forward: 5′-CCTTAGGAAGTTGCATTATGCTG; Reverse: 5′-ATTTATGAATTGAAAAGTTCAAGCAA). One of the papaya BAC end sequences was used to amplify a positive control for PCR of sex-specific repeats using the following primers: Foward 5′-TGACTCCATTGCCTGAATTTT-3′, and Reverse5′-TCCTCTCCATACCTTCTCGTG-3′.

### RT-PCR analysis

Total RNAs were extracted from samples (SunUp female, hermaphrodite plant, seeds and half ripen fruit, and AU9 female and male plant leaf) using the hot phenol extraction method (Sambrook et al., 1989). The cDNA was synthesized using SuperScript II™ reverse transcriptase according to the manufacturer’s instructions (Invitrogen). The expression of the papaya zinc finger protein was examined by RT-PCR using the following primers: (F: 5′-CACTGGTTTTGCGGAAATTG; R: 5′-TGCACTTAGCATCATTGCAATG). As an internal control for RT-PCR analysis, papaya *Actin* gene was used [43].

### Mantel test

To examine relationships between physical distances and sequence identities among sex-specific repeats, all pairwise sequence identities were obtained from the clastalW2 online tool (http://www.ebi.ac.uk/Tools/msa/clustalw2) and all pairwise physical distances were calculated manually. The sequence identities and the physical distances were used for Mantel test implemented in Genetic Analysis in Excel (GenAlEx 6.5) program [44]. Briefly, the sequence identities were manually arranged to Y matrix and the physical distances to X matrix as described in GenAlEx Tutorial 3. Then, Mantel test was performed with default set except for permutations of 9,999.

## Electronic supplementary material

Additional file 1: **Note GenBank accession numbers of new repeats identified from the sex determining region of papaya sex chromosomes.** (DOCX 12 KB)

Additional file 2: Table S1: Blast result of newly identified repeats against the sex determining region and papaya genome. (XLSX 13 KB)

Additional file 3: Table S2: Blast result of sex-specific repeats against the HSY and the corresponding X. (XLSX 36 KB)

Additional file 4: Figure S1: Pairwise sequence identities among different copies of each sex-specific repeat, (A) HSY-R29, (B) HSY-R162, or (C) X-R55 were plotted according to their physical distance. (DOCX 29 KB)
