# Supplementary material for: Accumulation of interspersed and sex-specific repeats in the non-recombining region of papaya sex chromosomes
Source: BMC Genomics. 2014 May 4;15(1):335. doi: 10.1186/1471-2164-15-335 (PMC4035066; doi:10.1186/1471-2164-15-335)
Supplement: Supplementary file 1 — Additional file 1: Note GenBank accession numbers of new repeats identified from the sex determining region of papaya sex chromosomes. (DOCX 12 KB) [file 12864_2013_6027_MOESM1_ESM.docx]

**Note 1**: GenBank accession numbers of new repeat sequences identified from the sex determining region of papaya sex chromosomes.

**Repeat ID Accession no.**

X-R55 KJ410362

HSY-R29 KJ410363

HSY-R162 KJ410364

RHSY15_11 KJ410365

RHSY15_58 KJ410366

RHSY15_171 KJ410367

RHSY15_213 KJ410368

RHSY15_216 KJ410369

RHSY15_229 KJ410370

RHSY15_29 KJ410371

RHSY15_326 KJ410372

RHSY15_56 KJ410373

RHSY15_568 KJ410374

RHSY15_65 KJ410375

RHSY15_97 KJ410376

RHSY15_136 KJ410377

RHSY15_57 KJ410378

RHSY15_253 KJ410379

RHSY15_92 KJ410380

RHSY15_40 KJ410381

RHSY15_201 KJ410382

RX15_17 KJ410383

RHSY15_91 KJ410384

RHSY15_46 KJ410385

RHSY15_315 KJ410386

RHSY15_558 KJ410387

RHSY15_262 KJ410388

RHSY15_150 KJ410389

RHSY15_76 KJ410390

RHSY15_36 KJ410391

RHSY15_128 KJ410392

RHSY15_27 KJ410393

RHSY15_55 KJ410394

RX15_10 KJ410395

RHSY15_80 KJ410396

RHSY15_48 KJ410397
