# Supplementary material for: Accumulation of interspersed and sex-specific repeats in the non-recombining region of papaya sex chromosomes
Source: BMC Genomics. 2014 May 4;15(1):335. doi: 10.1186/1471-2164-15-335 (PMC4035066; doi:10.1186/1471-2164-15-335)
Supplement: Supplementary file 4 — Additional file 4: Figure S1: Pairwise sequence identities among different copies of each sex-specific repeat, (A) HSY-R29, (B) HSY-R162, or (C) X-R55 were plotted according to their physical distance. (DOCX 29 KB) [file 12864_2013_6027_MOESM4_ESM.docx]

**Figure S1**. Pairwise sequence identities among different copies of each sex-specific repeat, (A) HSY-R29, (B) HSY-R162, and (C) X-R55 were plotted according to their physical distance. Mantel test implemented in GenAlEx6.5 was used for statistical analysis. Rxy (correlation coefficient of Mantel test) and one-tailed P-value (rxy-rand >= rxy-data) were 0.014 and 0.511 for HSY-R29, -0.033 and 0.378 for the HSY-R162, and -0.006 and 0.385 for X-R55, respectively.

**A**

**B**

**C**
